# Assessing reporting quality of randomized controlled trial abstracts in psychiatry: Adherence to CONSORT for abstracts: A systematic review

**DOI:** 10.1371/journal.pone.0187807

**Published:** 2017-11-08

**Authors:** Seung Yeon Song, Boyeon Kim, Inhye Kim, Sungeun Kim, Minjeong Kwon, Changsu Han, Eunyoung Kim

**Affiliations:** 1 Department of Health, Social and Clinical Pharmacy, College of Pharmacy, Chung-Ang University, Seoul, South Korea; 2 The Graduate School of Pharmaceutical Industry Management, Chung-Ang University, Seoul, South Korea; 3 Mine-Medical Clinical Research Lab, Korea University College of Medicine, Seoul, South Korea; University of Pennsylvania, UNITED STATES

## Abstract

**Background:**

Reporting quality of randomized controlled trial (RCT) abstracts is important as readers often make their first judgments based on the abstracts. This study aims to assess the reporting quality of psychiatry RCT abstracts published before and after the release of Consolidated Standards of Reporting Trials for Abstracts (CONSORT-A) guidelines.

**Methods:**

MEDLINE/PubMed search was conducted to identify psychiatric RCTs published during 2005–2007 (pre-CONSORT) and 2012–2014 (post-CONSORT). Two independent reviewers assessed abstracts using a 18-point overall quality score (OQS) based on the CONSORT-A guidelines. Linear regression analysis was conducted to analyze factors associated with reporting quality.

**Results:**

Among 1,927 relevant articles, 285 pre-CONSORT and 214 post-CONSORT psychiatric RCT abstracts were included for analysis. The mean OQS improved from 6.9 (range: 3–13; 95% confidence interval (CI): 6.7–7.2) to 8.2 (range: 4–16; 95% CI: 7.8–8.5) after the CONSORT-A guidelines. Despite improvement, methods of randomization, allocation concealment, and funding source remained to be insufficiently reported (<5%) even after the release of CONSORT-A. High-impact general medical journals, multicenter design, positive outcome, and structured abstracts were associated with better reporting quality.

**Conclusions:**

The reporting quality in psychiatric RCT abstracts, although improved, remains suboptimal. To improve reporting quality of psychiatry RCT abstracts, greater efforts by both investigators and journal editors are required to enhance better adherence to the CONSORT-A guidelines.

## Introduction

The reporting quality of RCT abstracts is important as the readers often make their initial assessment of articles based on the abstracts. In addition, due to the high volume of annual publications, as well as limited time and resources, clinicians may even make clinical decisions based solely on the information provided in the abstracts.

Recognizing the importance of well-informed abstracts and the need for improvements in the reporting of abstracts, the Consolidated Standards of Reporting Trials for Abstracts (CONSORT-A) was developed and finalized in January 2008. Many health care journals endorse the use of the CONSORT-A to provide guidance to authors about the necessary details and clarity required for reporting in abstracts [1]. However, low endorsement rates of editorial policies, including CONSORT, have been found within psychiatry journals [2, 3]. Han et al. (2009) study, which used the CONSORT statement 2001 intended for full articles, found that the reporting quality of psychiatry RCTs, although improved, remained suboptimal even after the release of the statement [4].

To our knowledge, no studies have assessed the reporting quality of RCT abstracts in the field of psychiatry. Thus, this study aims to assess the overall reporting quality of psychiatry RCT abstracts published before and after the release of the CONSORT-A, and to determine the trial characteristics associated with reporting quality.

## Materials and methods

### Study selection

We conducted a MEDLINE/PubMed search to identify all psychiatry RCTs from the top 20 psychiatry journals with the highest impact factor and four high-impact general medical journals with a broad readership (British Medical Journal [BMJ], Journal of the American Medical Association [JAMA], Lancet, and the New England Journal of Medicine [NEJM]), published within the periods of interest (01/01/2005 to 31/12/2007 and 01/01/2012 to 31/12/2014). The study periods were divided into pre-CONSORT-A (2005–2007) and post-CONSORT-A (2012–2014) periods for comparison. A lag time of 24 months was considered to accommodate a possible lag time between the publication of the CONSORT for Abstracts and the uptake of the recommendations.

### Inclusion and exclusion criteria

All abstracts of RCTs published in English and conducted on human subjects were included. A study was defined as an RCT if the participants were assigned to interventions that were described as random, randomly allocated, randomized, or if randomization was mentioned, and if a control group was included. The control group could receive a placebo, usual care, or a comparator. All other study designs, such as non-randomized studies, follow-up studies of previously published trials, studies analyzing more than two trials, crossover studies, diagnostic tests and biomarker analyses, economic analyses, safety analyses, reviews, protocols, editorials, and letters, were excluded.

### Data collection

Extracted data included the year of publication, target disease, intervention type (‘pharmacological’, ‘psychological treatment’, or ‘other’ e.g., electroconvulsive therapy), name of the intervention, name of the journal, impact factor of the journal, number of authors, funding source, region of publication, number of conducting centers (single or multicenter), trial outcomes (positive, negative, or unclear), abstract format (structured or unstructured), sample size, as well as journal CONSORT endorsement, and word count restriction. The target diseases were classified in accordance with the Diagnostic and Statistical Manual of Mental Disorders (DSM-5) [5]. With regard to trial outcomes, a trial was defined as positive when the experimental arm was considered superior to the standard arm in superiority trials, not inferior in non-inferiority trials, or equivalent in equivalence trials. Beyond this, all other studies were defined as trials with unclear outcomes. The region of publication was determined from the address of the first authors’ institution.

The CONSORT-A guideline was developed in 2008 and provides a list of essential items that authors should consider when reporting the abstracts of RCTs (http://www.consort-statement.org/extensions?ContentWidgetId=562). The CONSORT-A checklist items include identification of the study as randomized, trial design, participants, interventions, details of the trial’s objectives, clearly defined primary outcome, methods of randomization, blinding, number of participants randomized and analyzed in each group, the effect of interventions on primary outcomes and harms, trial conclusions, trial registration, and funding. Two reviewers independently assessed the adherence to the CONSORT-A guidelines by assigning ‘yes’ or ‘no’ to each item on the checklist. Both reviewers underwent training in evaluating RCTs using the CONSORT checklist, and the definition of each checklist item was discussed before the study was conducted. A pilot study was performed with randomly selected abstracts to assess inter-observer agreement using Cohen’s kappa statistics, and to resolve any discrepancies in the data extraction process.

### Rating of overall reporting quality

The overall quality score (OQS) was adopted from the methodology used by previous studies.[6–11] The OQS consists of 18 items modified from the CONSORT-A guidelines. Each item was given equal weight and a score of one. OQS% was then calculated by dividing the number of items met by the number of total items to generate a percentage score for easier interpretation and comparison with the results of previously published studies in the field.

### Statistical analysis

Descriptive statistics were performed on the characteristics of RCT articles in pre-COSORT-A and post-CONSORT-A periods. The overall number and proportion (%) of RCT abstracts that met each of the CONSORT for Abstracts checklist items were determined. Pearson chi-square analyses or Fisher exact tests, where applicable, were conducted for each of the CONSORT items to compare pre- and post-CONSORT-A RCT abstracts. A mean OQS was generated for each RCT abstract on a scale of 0 to 18 and a mean OQS% was calculated along with the 95% confidence interval (CI). A t-test was performed to compare the mean OQS% of article characteristics between pre-CONSORT-A and post-CONSORT-A periods. To analyze factors associated with higher OQS%, a linear regression analysis was performed. All tests for statistical significance were two-tailed, with the threshold set at 0·05. All analyses were performed using SPSS software (version 23·0; IBM Corporation, NY, USA).

## Results

**Fig 1:**
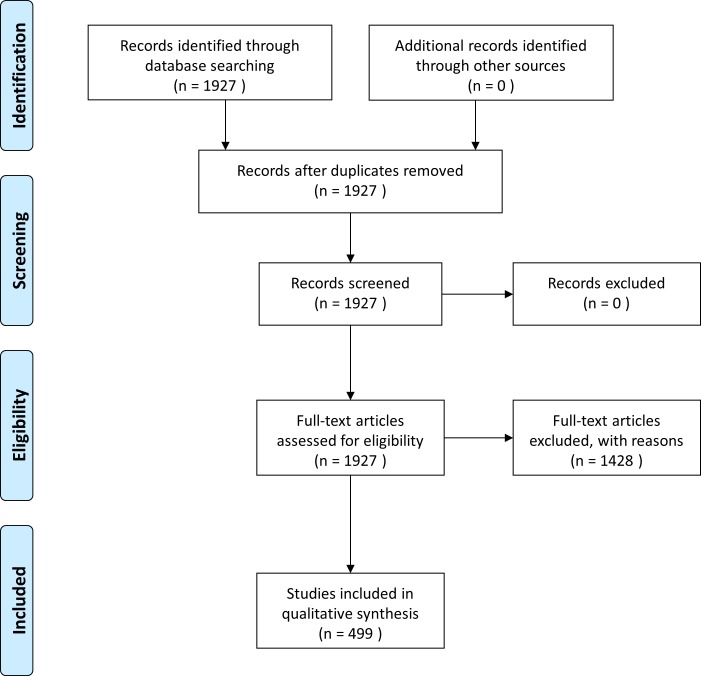
Search strategy and study selection.

Our search strategy identified 1,136 pre-CONSORT-A and 791 post-CONSORT-A RCT abstracts. After exclusion, 285 pre-CONSORT-A and 214 post-CONSORT-A RCT abstracts were included for analysis (Fig 1). The majority of pre-CONSORT-A RCTs (23·5%) addressed ‘schizophrenia and psychotic disorders’ as their target disease, while most post-CONSORT-A RCTs (19·6%) addressed ‘depressive disorders.’ The highest number of both pre- and post-CONSORT-A RCTs was published in the *Journal of Clinical Psychiatry* (31·2% and 29·0%, respectively; Table 1). Although the top 20 high-impact psychiatry journals were included in the initial search, abstracts from 18 psychiatry journals were finally included.

**Table 1:** Trial characteristics of included abstracts.

Characteristics	Pre- CONSORT-A (n = 285)		Post- CONSORT-A (n = 214)	
	N	%	N	%
**Target disease^a^**				
Schizophrenia and psychotic disorders	67	23·5	37	17·3
Depressive disorders	54	18·9	42	19·6
Anxiety disorders	31	10·9	18	8·4
Bipolar and related disorders	28	9·8	30	14·0
Substance use and addictive disorders	20	7·0	13	6·1
Feeding and eating disorders	12	4·2	6	2·8
Trauma and stressor-related disorders	11	3·9	11	5·1
Obsessive-compulsive and related disorders	8	2·8	5	2·3
Others	54	18·9	52	24·3
Intervention***				
Pharmacological	185	64·9	112	52·3
Psychological	58	20·4	44	20·6
Pharmacological and psychological	23	8·1	14	6·5
Others	19	6·7	44	20·6
Journal***				
Arch Gen Psychiatry^b^	25	8·8	6	2·8
J Clin Psychiatry	89	31·2	62	29·0
Neuropsychopharmacology	8	2·8	8	3·7
Acta Psychiatr Scand	6	2·1	3	1·4
Psychol Med	10	3·5	7	3·3
Br J Psychiatry	27	9·5	21	9·8
Am J Psychiatry	50	17·5	33	15·4
Biol Psychiatry	33	11·6	11	5·1
BMJ	6	2·1	1	0·5
JAMA	4	1·4	6	2·8
Lancet	0	0·0	5	2·3
N Engl J Med	2	0·7	2	0·9
Others	25	8·8	49	22·9
Impact factor**				
Less than 5	10	3·5	16	7·5
5–10	155	54·4	134	62·6
More than 10	120	42·1	64	29·9
**Number of Authors**				
Less than 4	28	9·8	14	6·5
4–7	131	46·0	96	44·9
More than 7	126	44·2	104	48·6
Funding source***				
Government/peer reviewed/cooperative groups	143	50·2	163	76·2
Industry	88	30·9	29	13·6
Both	14	4·9	15	7·0
No funding or none reported	40	14·0	7	3·3
**Region of publication**				
Europe	76	26·7	73	34·1
North America	167	58·6	113	52·8
Asia	23	8·1	18	8·4
Others	19	6·7	10	4·7
**Centers**				
Single center	140	49·1	100	46·7
Multicenter	145	50·9	114	53·3
**Trial outcome**				
Positive	167	58·6	118	55·1
Negative	96	33·7	76	35·5
Unclear	22	7·7	20	9·3
Abstract structure**				
Structured	267	93·7	184	86·0
Unstructured	18	6·3	30	14·0
**Sample size**				
Median (interquartile range)	106 (50–254)		106 (60–209·25)	
**CONSORT endorsement**				
Yes	251	88·1	180	84·1
No	34	11·9	34	15·9
**Word count limit**				
<250	33	11·6	24	11·2
≥250 or no word limit	252	88·4	190	88·8

^a^ Classified in accordance with the Diagnostic and Statistical Manual of Mental Disorders (DSM-5).

^b^ Arch Gen Psychiatry was renamed JAMA psychiatry in 2013.

*P<0.05

**P<0.01

***P<0.001.

χ^2^ test or Fisher’s exact tests performed between pre-CONSORT-A (2005–2007) and post-CONSORT-A (2012–2014)

### Reporting of general items

**Fig 2:**
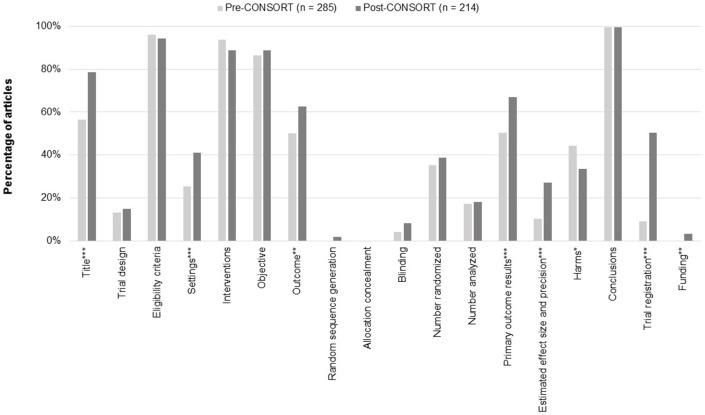
Reporting of CONSORT for abstract checklist items. *P<0.05; **P<0.01; ***P<0.001.

The quality assessment of psychiatry RCT abstracts is shown in Fig 2. A significantly greater number of studies in the post-CONSORT-A period stated ‘randomized’ in the title compared to the pre-CONSORT-A period (56·5% vs. 78·5%; *P<*0.001). The trial design was described by only a small portion of the abstracts in both the pre-CONSORT-A (13·3%) and post-CONSORT-A (15·0%) periods.

### Reporting of trial methodology

Nearly all studies reported the eligibility criteria for participants, with 96.1% of studies reporting it in the pre-CONSORT-A period and 94·4% in the post-CONSORT-A period. Reporting on the specific setting of data collection was relatively low; however, its reporting frequency increased significantly in the post-CONSORT-A period (25·3% vs. 41·1%; *P<*0.001). A majority of RCT abstracts reported on the interventions assigned for each group (pre-CONSORT-A, 93·7%; post-CONSORT-A, 88·8%), and defined a specific objective or hypothesis (pre-CONSORT-A, 86·3%; post-CONSORT-A, 88·8%). More than half of the abstracts defined the primary outcome, and reporting on this criterion significantly increased over each period (50·5% vs. 62·6%; *P =* 0·007). Few abstracts reported on the method of randomization (pre-CONSORT-A, 0·0%; post-CONSORT-A, 1·9%), and there were no reports on allocation concealment. Blinding details regarding participants were also rarely reported (pre-CONSORT-A, 4·2%; post-CONSORT-A, 8·4%), and 59·6% of pre-CONSORT-A and 46·3% of post-CONSORT-A studies referred to blinding methods using words such as ‘single’ or ‘double’.

### Reporting of trial results

The number of participants randomized (pre-CONSORT-A, 35·4%; post-CONSORT-A, 38·8%) and the number analyzed (pre-CONSORT-A, 17·2%; post-CONSORT-A, 18·2%) were inadequately reported by studies from both periods. Reporting on trial outcomes significantly improved with the reporting of primary outcome results increasing from 50.5% pre-CONSORT-A to 66·8% post-CONSORT-A (*P<*0·001), and details regarding effect size and precision also increased from 10·2% to 27·1% (*P<*0·001). Interestingly, reporting on harm was found to be higher before the publication of the CONSORT statement (pre-CONSORT-A, 44·2%; post-CONSORT-A, 33·6%; *P =* 0·017).

### Reporting of trial conclusions

Almost all of the studies reported conclusions in both the pre- and post-CONSORT-A periods (pre-CONSORT-A, 99·6%; post-CONSORT-A, 99·5%). Reporting on trial registration significantly improved from 9·1% pre-CONSORT-A to 50·5% post-CONSORT-A (*P*<0·001). Reporting on the funding source also improved from no studies in the pre-CONSORT-A period to 3·3% of studies in the post-CONSORT-A period reporting funding (*P =* 0·003).

### OQS%

The mean OQS on a 0 to 18 scale was 6·9 (range: 3–13; 95% CI: 6·7–7·2) for pre-CONSORT-A and 8·2 (range: 4–16; 95% CI: 7·8–8·5) for post-CONSORT-A studies. The mean OQS% improved significantly from 38·4% (95% CI: 37·0–39·8) to 45·4% (95% CI: 43·5–47·3) after the publication of CONSORT for Abstracts (Table 2). The mean OQS% was higher in the abstracts in each kind of interventions; however, significant improvements were not observed in those with both pharmacological and psychological interventions. The highest mean OQS% was observed in the high-impact general medical journals (69·0%; 95% CI: 61·5–76·6). The mean OQS% for journals with an impact factor of five and over increased significantly (between 5 and 10: 45·9%; 95% CI: 43·8–47·9; more than 10: 47·0%; 95% CI: 42·9–51·2) while those with an impact factor of less than five did not show significant improvement (35·1%; 95% CI: 29·6–40·6). In addition, structured abstracts indicated a significant improvement in mean OQS% (46·9%; 95% CI: 44·9–48·9). The improvements in abstract reporting did not depend on CONSORT endorsements, as significant improvement was observed both with endorsements (45·9%; 95% CI: 43·9–47·9) and without (45·4%; 95% CI: 40·7–50·1). The annual mean OQS% for abstracts from high-impact general medical journals showed a tendency to improve in the post-CONSORT-A period (2012, 55·6% [95% CI: 45·3, 65·8]; 2013, 66·7% [95% CI: 56·5, 76·9]; 2014, 76·2% [95% CI: 69·3, 83·0]). However, the annual mean OQS% for abstracts from psychiatry journals did not improve and was stagnant (2012, 45·1% [95% CI: 42·4, 47·7]; 2013, 44·8% [95% CI: 41·9, 47·7]; 2014, 40·8% [95% CI: 37·4, 44·3]).

**Table 2:** Mean overall quality score (OQS) on a modified percentage scale according to the characteristics of the included psychiatry RCT abstracts.

Characteristics	Pre-CONSORT-A, mean OQS% (95% CI)	Post-CONSORT-A, mean OQS% (95% CI)
**Year of publication**	38·4% (37·0–39·8)	45·4% (43·5–47·3)***
**Target disease^a^**		
Schizophrenia and psychotic disorders	35·5% (32·8–38·1)	47·1% (41·6–52·7)***
Depressive disorders	41·5% (38·4–44·5)	48·7% (44·1–53·2)**
Anxiety disorders	36·9% (32·4–41·4)	39·2% (32·9–45·5)
Bipolar and related disorders	43·7% (38·6–48·7)	42·6% (38·2–47·0)
Substance use and addictive disorders	43·4% (38·7–48·0)	42·7% (33·9–51·6)
Feeding and eating disorders	39·4% (29·0–49·7)	50·9% (30·9–70·9)
Trauma and stressor-related disorders	39·4% (32·2–46·6)	40·4% (32·4–48·4)
Obsessive-compulsive and related disorders	29·2% (21·0–37·3)	42·2% (25·6–58·8)
Others	36·3% (33·0–39·6)	46·7% (43·2–50·2)***
**Intervention**		
Pharmacological	40·0% (38·3–41·8)	46·6% (44·1–49·1)***
Psychological	33·2% (30·5–36·0)	43·2% (39·8–46·6)***
Pharmacological and psychological	40·3% (34·1–46·6)	46·0% (38·0–54·1)
Others	36·5% (32·8–40·3)	44·4% (38·9–50·0)*
**Journal**		
High-impact general medical journals^b^	55·6% (46·7–64·5)	69·0% (61·5–76·6)*
Arch Gen Psychiatry^c^	47·3% (44·3–50·4)	57·4% (47·2–67·6)**
J Clin Psychiatry	42·3% (40·0–44·6)	53·1% (50·9–55·4)***
Neuropsychopharmacology	31·9% (20·4–43·5)	43·8% (34·0–53·5)
Acta Psychiatr Scand	36·1% (26·5–45·7)	42·6% (10·7–74·5)
Psychol Med	35·0% (28·2–41·8)	42·1% (35·5–48·6)
Br J Psychiatry	31·3% (27·4–35·1)	42·1% (36·7–47·4)**
Am J Psychiatry	37·3% (34·5–40·2)	39·6% (35·9–43·3)
Biol Psychiatry	31·3% (27·9–34·7)	35·9% (28·7–43·0)
Others	31·1% (26·9–35·4)	35·8% (32·5–39·2)
**Impact factor**		
Less than 5	31·1% (24·5–37·7)	35·1% (29·6–40·6)
5–10	38·0% (36·2–39·9)	45·9% (43·8–47·9)***
More than 10	39·6% (37·4–41·8)	47·0% (42·9–51·2)*
**Number of authors**		
Less than 4	35·5% (31·7–39·3)	40·5% (34·2–46·7)
4–7	36·3% (34·3–38·4)	44·0% (41·6–46·5)***
More than 7	41·3% (39·2–43·4)	47·3% (44·3–50·3)**
**Funding source**		
Government/peer reviewed/cooperative groups	37·5% (35·4–39·5)	43·9% (41·7–46·1)***
Industry	41·8% (39·3–44·3)	51·3% (47·4–55·3)***
Both	36·9% (30·4–43·4)	54·4% (47·3–61·6)**
No funding or none reported	35·1% (31·7–38·6)	36·5% (27·7–45·3)
**Region of publication**		
Europe	36·9% (34·2–39·6)	45·7% (42·3–49·1)***
North America	39·4% (37·6–41·3)	45·6% (43·1–48·1)***
Asia	38·9% (33·1–44·7)	45·1% (36·1–54·0)
Others	35·4% (30·4–40·4)	41·1% (34·1–48·2)
**Centers**		
Single center	34·5% (32·7–36·3)	40·0% (37·5–42·5)***
Multicenter	42·3% (40·3–44·2)	50·1% (47·6–52·7)***
**Trial outcome**		
Positive	40·0% (38·2–41·8)	47·9% (45·3–50·5)***
Negative	37·6% (35·3–34·0)	44·2% (41·2–47·1)**
Unclear	30·1% (24·7–35·4)	35·3% (29·1–41·4)
**Abstract structure**		
Structured	38·9% (37·5–40·4)	46·9% (44·9–48·9)***
Unstructured	31·2% (25·0–37·3)	36·3% (31·9–40·7)
**Sample size**		
≤100	35·0% (33·1–36·9)	42·1% (39·7–44·4)***
>100	41·6% (39·7–43·5)	48·3% (45·5–51·1)***
**CONSORT endorsement**		
Yes	39·1% (37·6–40·6)	45·9% (43·9–47·9)***
No	38·4% (34·4–42·4)	45·4% (40·7–50·1)*
**Word count limit**		
<250	32·2% (29·0–35·6)	42·1% (37·7–46·8) *
≥250 or no word limit	39·3% (37·7–40·8)	45·8% (43·7–47·8) ***

OQS, Overall Quality Score.

^a^ Classified in accordance with the Diagnostic and Statistical Manual of Mental Disorders (DSM-5).

^b ^BMJ, JAMA, N Engl J Med, and Lancet.

^c^ Arch Gen Psychiatry was renamed JAMA psychiatry in 2013.

*P<0.05

**P<0.01

***P<0.001

t-tests performed between pre-CONSORT-A (2005–2007) and post-CONSORT-A (2012–2014)

### Factors associated with reporting quality

Table 3 shows the results of the linear regression analysis. The univariate analysis showed that abstracts published in high-impact general medical journals, with an impact factor of 5 to 10, or greater than 10, and with CONSORT endorsement, were more likely to have better reporting quality. In addition, those with industry or both industry and non-industry funding, a multicenter design, positive or negative outcomes, a structured format, and large sample size were more likely to have a higher mean OQS%. In the multiple linear regression model, high-impact general medical journals, number of authors greater than 7, a multicenter design, positive or negative outcomes, a structured abstract, and word count limit greater than 250 or no word limit, were associated with better reporting quality. On the other hand, studies with psychological interventions were associated with lower reporting quality compared to studies with pharmacological intervention.

**Table 3:** Linear regression derived estimates and 95% CI with mean overall quality score on a modified percentage scale as the dependent variable for psychiatry RCT abstracts.

Characteristics	Univariate analysis, estimate 95% CI	Multivariate analysis, estimate 95% CI
**Year of publication**		
Pre-CONSORT	Reference	Reference
Post-CONSORT	6.96 (4.66, 9.27)***	7.3 (5.2, 9.4)***
**Intervention**		
Pharmacological	Reference	Reference
Psychological	-3·4 (-8·34, 1·55)	-4.74 (-7.75, -1.72)**
Pharmacological and psychological	-0·55 (-8·43, 7·33)	-3.16 (-6.47, 0.14)
Others	-2·13 (-7·08, 2·81)	-0.8 (-4.71, 3.1)
**Journal type**		
High-impact general medical journals ^a^	Reference	Reference
Psychiatry journals ^b^	-25·3 (-32·18, -18·42)***	-19.94 (-24.59, -15.28)***
**Impact factor**		
Less than 5	Reference	Reference
5–10	10·78 (3·58, 17·99)**	3.31 (-2.61, 9.23)
More than 10	11·98 (4·37, 19·59)**	-2.32 (-9, 4.35)
**Number of authors**		
Less than 4	Reference	
4–7	3·56 (-4·32, 11·45)	2.96 (-0.63, 6.55)
More than 7	6·85 (-1, 14·7)	5.21 (1.52, 8.9)**
**Funding source**		
No funding or none reported	Reference	Reference
Government/peer reviewed/cooperative groups	7·39 (-2·98, 17·76)	-0.18 (-3.87, 3.5)
Industry	14·83 (3·52, 26·15)*	2.21 (-1.87, 6.29)
Both	17·94 (5·64, 30·23)**	3.12 (-2.18, 8.41)
**Region of publication**		
Europe	Reference	Reference
North America	-0·68 (-8·01, 6·66)	-0.05 (-2.46, 2.37)
Asia	-0·11 (-4·3, 4·07)	1.75 (-2.12, 5.62)
Others	-4·63 (-14·03, 4·77)	0.29 (-4.11, 4.68)
**Centers**		
Single center	Reference	Reference
Multicenter	10·15 (6·59, 13·7)***	3.7 (1.24, 6.16)**
**Trial outcome**		
Unclear	Reference	Reference
Positive	12·65 (6·15, 19·15)***	7.86 (4.22, 11.5)***
Negative	8·87 (2·12, 15·63)*	5.96 (2.2, 9.71)**
**Abstract structure**		
Unstructured	Reference	Reference
Structured	10·59 (5·31, 15·88)***	9.08 (4.17, 13.99)***
**Sample size**		
≤100	Reference	Reference
>100	6·29 (2·57, 10)**	1.96 (-0.45, 4.38)
**CONSORT endorsement**		
No	Reference	Reference
Yes	5·8 (0·66, 10·94)*	-0.19 (-3.58, 3.21)
**Word count limit**		
< 250	Reference	Reference
≥250 or no word limit	0·51 (-3·57, 4·59)	3.14 (0.22, 6.05)*

^a ^BMJ, JAMA, N Engl J Med, and Lancet.

^b ^Acta Psychiatr Scand, Addiction, Am J Psychiatry, Arch Gen Psychiatry (renamed JAMA psychiatry in 2013), Biol Psychiatry, Bipolar Disord, Br J Psychiatry, Eur Neuropsychopharmacol, J Child Psychol Psychiatry, J Clin Psychiatry, J Neurol Neurosurg Psychiatry, J Psychiatry Neurosci, Int J Neuropsychopharmacol, Neuropsychopharmacology, Psychol Med, Psychoneuroendocrinology, Psychother Psychosom, Schizophr Bull.

*P<0.05

**P<0.01

***P<0.001.

## Discussion

Randomized controlled trials (RCTs) are the gold standard for clinical trials, providing the most credible evidence for intervention efficacy, and are major sources of evidence-based research. Accurate and complete reporting of trial results is essential for readers to understand how a clinical trial was conducted and judge its validity. Reporting quality of randomized controlled trial (RCT) is also important especially in light of new NIH guidelines on rigor, reproducibility, and transparency [12]. As a field, psychiatry can only be taken more seriously with better reporting of outcomes and better transparency on trial design. Although reporting quality itself does not necessarily correlate with study quality, inadequate reporting has the potential to bias the estimates of treatment effects in RCTs [7, 13]. The reporting quality of RCT abstracts is also of great importance because the readers often make decisions to read the full article based on the abstracts.

The reporting quality of psychiatry RCT abstracts improved significantly after the publication of the CONSORT for Abstracts. However, despite improvement, the reporting quality of abstracts remains suboptimal with a post-CONSORT-A mean OQS% of 45·4%. Similar studies. reported a mean OQS% of 58·6–62·5% in the field of dentistry, and an annual increase in mean OQS% to more than 50% after the publication of the CONSORT for Abstracts in oncology [6, 14, 15]. The main finding of our study is that articles published in medical journals and studies with pharmacological intervention have better adherence to CONSORT for Abstract guidelines than psychiatry-based journals and studies with non-pharmacological intervention. Thus, although significant increase in the reporting quality of psychiatry abstracts was noted, it is still not impressive. In our study, abstracts published in high-impact general medical journals, including BMJ, JAMA, Lancet, and NEJM, showed a much higher mean OQS% of 69·0% after the release of the CONSORT for Abstracts compared to those published in psychiatry journals, with OQS% of 43.8%. This may be explained by the higher endorsement of editorial policies including the CONSORT guidelines by journals, and the rigorous peer review process conducted before publication. Interestingly, impact factor was not a significant factor affecting abstract reporting quality, while high-impact general medical journal type was. This shows that more efforts are required to endorse and promote adherence to CONSORT guidelines within psychiatry journals. The endorsement rates for psychiatry journals are reported to be low, and of the 18 high-impact psychiatry journals included in our study, only 12 journals endorsed CONSORT, with a 66·7% endorsement rate [2].

Overall, less than a quarter of the included abstracts reported on the trial design, method of randomization, allocation concealment, blinding, and sources of funding. Such results are consistent with previous findings [6, 16, 17]. Of note, CONSORT items, including method of randomization, allocation concealment, and source of funding, were not reported at all in the pre-CONSORT-A period, and even after the CONSORT-A statement they were reported in less than 5% of the abstracts. Prior studies have found inadequate reporting of allocation concealment to be associated with exaggerated treatment effects [18, 19]. It must be noted that a lack of description of important methodological items could cause bias and influence the internal validity of the trial [14]. In addition, according to the CONSORT guidelines the funding source is an important piece of information for the reader and should be reported in abstracts [20]. Our study found very few psychiatry RCT abstracts that reported funding source information, although it should be clearly stated in the abstract.

The use of a structured abstract was associated with better abstract reporting quality, which is in agreement with the findings of previous studies [6, 21]. Structured abstracts can improve readability, and facilitate an easy assessment of the information reported in the abstract. Unfortunately, over 30% of the psychiatry journals included in this analysis did not recommend a structured abstract format, even after the publication of the CONSORT for Abstracts. Therefore, psychiatry journals need to actively recommend the use of structured abstracts, as it can improve the quality of reporting in abstracts. Also, 250–300 words are considered sufficient to address all items of CONSORT for Abstracts [7, 19]. Our results support this with abstracts published in journals with word count limit greater than 250 or no word limit at all, achieving higher adherence to the items. Similar to the study by Mbuagbaw et al., multicenter studies were found to have a better abstract reporting quality [22]. The exact reason behind such a phenomenon is unknown; however, it can be assumed that multicenter studies are of a larger scale and involve a greater number of researchers, possibly leading to better reporting of abstracts.

This study has several limitations. First, our study is not fully representative of all published psychiatry RCTs. This is because we extracted only certain abstracts from psychiatry journals, and excluded studies with primary outcomes other than changes in clinical symptoms for psychiatric diseases, such as studies on genetic psychiatry and abnormal morphology of the brain. However, our results may sufficiently reflect the overall trends in the abstract reporting of psychiatry RCTs because our study included the top 20 psychiatry journals. Second, our study analyzed the adequacy of reporting based on the CONSORT checklist, without considering whether the content of the full article was accurately reflected in the abstract. This was beyond the scope of our study. Thus, further research that assesses the accuracy of reporting in abstracts is needed. Finally, the type of intervention could have affected our findings. In general, in studies with non-pharmacological treatments, it is difficult to provide a sham intervention and often impossible to blind patients and care providers [23]. Accordingly, extended abstract reporting guidelines for studies with non-pharmacological interventions are required to improve the quality of reporting.

In conclusion, this study demonstrated that the quality of reporting in psychiatry RCT abstracts, although improved, remains suboptimal after the publication of the CONSORT guidelines. In particular, the finding of inadequate reporting on important methodological items could result in biased interpretations. Based on our findings, health professionals and policymakers should be careful when interpreting the information reported in psychiatry RCT abstracts. Moreover, additional efforts from both researchers and editors in the field of psychiatry appear to be necessary for better adherence to the CONSORT for Abstract guidelines and the provision of informative abstracts for readers.

## Supporting information

S1 Checklist PRISMA checklist. (DOC)

S1 Table Raw data references. (XLSX)
